# Exploring the Subjective Caregiving Experience Among Chinese, Korean, and Southeast Asian Communities

**DOI:** 10.1093/geroni/igab046.1741

**Published:** 2021-12-17

**Authors:** Sara Powers, Rachel Schaffer, David Bass, Ocean Le, Lauren Pongan

**Affiliations:** 1 Benjamin Rose Institute on Aging, Cleveland, Ohio, United States; 2 Diverse Elders Coalition, Diverse Elders Coalition, New York, United States

## Abstract

Although the Asian American community is one of the fastest growing racial groups in the US, members of this group continue to be underserved and understudied, especially when it comes to the needs of family caregivers. Therefore, through a national initiative to understand the lived experiences of diverse family and friend caregivers, survey data was collected from a variety of Asian American ethnic subgroups including Chinese (n=148), Korean (n=131), and Southeast Asian (i.e., Vietnamese, Hmong, Cambodian, Laotian; n=161). Surveys were distributed in-person and online, and also offered in the translated native languages of the abovementioned groups. Caregivers had to be 18 years and older and providing care to a person aged 55 and older who needed assistance because of ongoing health problems or disabilities. For the overall sample of Asian American caregivers (n=440), participants were on average 51.68 years of age (SD=15.98), identified as female (n=336), were not born in the US (n=348), lived with the care receiver (n=247), and reported less than $10,000 in income per year (n=199). As guided by the Stress Process Model and through a series of ANOVA tests, when compared on all major outcomes, Southeast Asian caregivers significantly reported: 1) more difficulty with care related tasks (e.g., financial/legal decisions), 2) a stronger cultural commitment to caregiving, 3) higher work strain, and 4) more depressive symptomology. Discussion will focus on opportunities for professionals to meet the needs of Asian American caregivers through the use of available trainings and programs aimed to support diverse caregivers.

